# Diabetes Mellitus in Children and Its Effect on Caregivers' Mental Health

**DOI:** 10.7759/cureus.2409

**Published:** 2018-04-02

**Authors:** Uzair Yaqoob, Mannan Ali Khan, Lavina Khemani, Faizan -ul-Haq, Javeryah Rafiq, Ayesha Syed Iftikhar

**Affiliations:** 1 Sindh Medical College, Dow University of Health Sciences, Karachi, PAK; 2 Baqai Medical University

**Keywords:** diabetes, depression, anxiety, caregiver health

## Abstract

Introduction

Caring for a child with diabetes often has a negative effect on the mental health of caregivers and other family members. The goal of this study was to determine the effect of caring for children with diabetes on the mental health of caregivers.

Materials and methods

This case-control study was conducted in the National Institute of Child Health from October to November 2017 to compare the mental health effects associated with caring for children with diabetes as against caring for children without diabetes. The case group consisted of the caregivers of 60 children with diabetes, and the control group consisted of the caregivers of 60 children without diabetes. A validated questionnaire with two scales was used. Frequencies, percentages, confidence intervals, and p-values were reported for the categorical variables. The variables were analyzed using Patient Health Questionnaire 9 (PHQ-9) and Generalized Anxiety Disorder 7 (GAD-7) scales to determine associations.

Results

In the case group, most caregivers were mothers, 60% had consanguineous marriages, 21.7% were employed, and 21.7% were suffering from a long-term illness. Of those reporting a disease, 13.3% reported a change in their disease. Most caregivers (71.7%) received family support, and 78.3% of caregivers had social support. Most caregivers in the case group had mild depression, and 35% had mild anxiety. In the control group, most caregivers were mothers, 5% were employed, and 35% had disease(s). Of those reporting a disease, 15% reported a change in their health. Most of the caregivers in the control group (93.3%) had family support, and 85% had social support. Most (62%) were found to have mild depression, and more than half had no anxiety. Most children in the control group were under five years old, and most children in the case group were over 10 years old. Nearly half of the children in the case group had a positive family history of thalassemia, and 68.3% of them have insulin administered once daily. Strong variables that affect caregiver mental health were caregiver age, health changes, and consanguinity.

Conclusion

The caregivers of children with diabetes have a higher incidence of anxiety compared with the caregivers of children without diabetes; depression was similar for both groups. Health care providers should be aware of the differences in parenting stress related to caring for children with long-term illnesses and should consider ways to help improve the well-being of these caregivers.

## Introduction

Diabetes mellitus is a group of metabolic diseases in which hyperglycemia results from either defective insulin secretion or insulin action or both. Chronic hyperglycemia results in the long-term dysfunction of various organs, especially the eyes, kidneys, nerves, heart, and blood vessels. The symptoms of marked hyperglycemia include polyuria, polydipsia, polyphagia, weight loss, increased susceptibility to infections, and occasional blurred vision [1]. Diabetes is generally classified into two types: insulin dependent and non-insulin dependent. Non-insulin dependent diabetes develops due to insulin resistance in obese people. A third type of diabetes, gestational diabetes mellitus, occurs only in pregnant women [2]. The incidence of chronic illnesses in children is rising; 7% to 18% of children in the United States are suffering from chronic illnesses [3]. These chronic illnesses affect not only the child but also the child’s entire family [4].

According to a study comparing the parents of children with different illnesses, parents of children with type 1 diabetes and asthma reported greater stress [5]. Stress in parents of children with diabetes is multifactorial, but it is directly linked to increased parental anxiety, decreased hope, and reduced feelings of self-efficacy [6].

There is a negative effect on the mental health of family members when there is a child with diabetes in the family. The goal of this research is to determine the effect of caring for children with diabetes on the mental health of caregivers.

## Materials and methods

This was a case-control study conducted from October to November 2017 in the outpatient department of the National Institute of Child Health. The case group consisted of 60 caregivers of children (up to 14 years old) with diabetes, and the control group consisted of 60 caregivers of children (up to 14 years old) without diabetes. Caregivers of children with other disorders were excluded. A validated questionnaire and two scales (i.e.,  PHQ-9 (Patient Health Questionnaire) and GAD-7 (Generalized Anxiety Disorder)) were used. The information collected included socio-demographic factors, factors related to the child’s disease, and lifestyle factors assessed via caregiver interviews. The questionnaires were completed by the principal investigator. Privacy was maintained, and the interviews were conducted in separate rooms. Participants were assured of confidentiality, that the information provided would be used for research purposes only, and that their identities will not be disclosed. The data were entered into spreadsheet software (Microsoft Excel 2010, Microsoft, Redmond, Washington, US) and then analyzed using SPSS for Windows, Version 16.0 (IBM, Armonk, New York, US).

The variables were analyzed against the PHQ-9 and GAD-7 score scales to assess the psychiatric status of the caregivers of the diabetic child [7].

The PHQ-9 scale is a multi-purpose instrument for screening, diagnosing, monitoring, and measuring the severity of depression. It has 61% sensitivity and 94% specificity in adults. It scores patients on a scale from zero to 27, where a score of zero to five indicates mild depression, six to ten moderate depression, 11 to 15 moderately severe depression, and 16 to 20 severe depression [8].

The GAD-7 scale is a self-reported questionnaire for screening the severity of the generalized anxiety disorder. It is 70% to 90% sensitive and 80% to 90% specific across disorders. It scores a patient from zero to 21 where a score of zero to five is no anxiety, five to nine is mild anxiety, 10 to 14 is moderate anxiety, and a score greater than 15 indicates severe anxiety [8].

## Results

Table 1 presents data on caregivers in the case group. In the case group, 60% of caregivers had consanguineous marriages, and 78.3% were women. Most caregivers (63.3%) were of 25 to 40 years old. Nearly all (98.3%) of them were married, and 90% were Muslims. Also, 21.7% of these caregivers were employed, and 76.7% of the caregivers were the mothers of the child with diabetes. Most caregivers reported having access to family and social support.

**Table 1:** Case group caregiver data

		Frequency	Percentage
Consanguinity	Yes	36	60
	No	24	40
Gender	Female	47	78.3
	Male	12	20
	Missing	1	1.7
Age (years)	<25	3	5
	25-40	38	63.3
	>40	14	23.3
	Missing	5	8.3
Marital status	Married	59	98.3
	Unmarried	1	1.7
Religion	Muslim	54	90.0
	Christian	6	10.0
Employment	Yes	13	21.7
	No	47	78.3
Relation	Mother	46	76.7
	Father	10	16.7
	Aunt	1	1.7
	Grandmother	1	1.7
	Uncle	2	3.3
Health status	No disease	47	78.3
	Had disease	13	21.7
Health changes	No change	52	86.7
	Change	8	13.3
Addiction	Yes	10	16.7
	No	50	83.3
Family support	Yes	43	71.7
	No	17	28.3
Social support	Yes	47	78.3
	No	13	21.7

Of the caregivers in the case group, 56% had mild depression, 42% had moderate depression, and 2% had severe depression according to PHQ-9 evaluation. The GAD-7 analysis revealed that 38% of caregivers in the case group had no anxiety, 35% had mild anxiety, 22% had moderate anxiety, and 5% had severe anxiety.

Table 2 presents information on the children with diabetes in the case group. Of those, 70% were younger than five years, and 25% were five to ten years old. Nearly half (53.3%) had a positive family history of diabetes. Most children in the case group receive insulin once per day, while 15% receive insulin twice per day. While most of the children in this group were diagnosed with diabetes at an age over 12 months, 16.7% were diagnosed at an age under six months.

**Table 2:** Case group children data

		Frequency	Percentage
Age (years)	<5	7	11.7
	5-10	26	43.3
	>10	27	45.0
Family history	Yes	32	53.3
	No	28	46.7
Order of sibling	1	25	41.7
	2	13	21.7
	3	9	15.0
	4	5	8.3
	5	5	8.3
	6	1	1.7
	7	1	1.7
	10	1	1.7
Insulin administration	Once a day	41	68.3
	Twice a day	9	15.0
	Thrice a day	1	1.7
	Four times a day	9	15.0
Age of diagnosis	<6 months	10	16.7
	6-12 months	8	13.3
	>12 months	42	70.0
Place of diagnosis	G.P	21	35.0
	Tertiary care	39	65.0

Table 3 presents data on caregivers in the control group. Five percent of caregivers in the control group were employed, 95% were unemployed. Also, 95% were mothers of the child, 65% reported that they do not have any disease, and of those who did report disease, 15% reported a change in their disease(s). Eighty-five percent had no change in their health, and 93.3% of the caregivers reported that they have family support while 85% of them said that they had social support.

**Table 3:** Control group caregiver data

		Frequency	Percentage
Gender	Female	60	100
	Male	-	-
Employment	Yes	3	5
	No	57	95
Relation	Mother	57	95
	Father	-	-
	Aunt	3	5
	Grandmother	-	-
	Grandfather	-	-
Health status	No disease	39	65
	Had disease	21	35
Health changes	No change	51	85
	Change	9	15
Family support	Yes	56	93.3
	No	4	6.7
Social support	Yes	51	85
	No	9	15

In the control group, 62% of caregivers scored mild depression in the PHQ-9 assessment, 25% scored moderate depression, 5% scored moderately severe depression, and 8% scored severe depression. Half of the caregivers in the control group had no anxiety per the GAD-7 analysis, while 38% were found to have mild anxiety, and 12% moderate anxiety. No caregivers in the control group scored for severe anxiety.

There was no significant difference in the incidence of depression between the case and control group caregivers (p = 0.149). However, the caregivers of children with diabetes were significantly more anxious than the caregivers of children without diabetes (p = 0.006).

Table 4 presents information on the children of the caregivers in the control group. Most of the children without diabetes were under age five, 25% were five to ten years old, and 10% were over the age of 10.

**Table 4:** Control group children data

		Frequency	Percentage
Age (years)	<5 years	39	65
	5-10 years	15	25
	>10 years	6	10

Table 5 presents the factors associated with anxiety and depression in caregivers of children with diabetes. Table 6 presents factors associated with anxiety and depression in caregivers of children without diabetes. We found a positive association between caregiver age and severe anxiety; two of the 14 caregivers in the case group over age 40 suffered from severe anxiety, while only one of the three caregivers in the case group under age 25 had severe anxiety. Of those aged 25 to 40 years (n = 38), none had severe anxiety. There is also an association between caregiver age and moderately severe depression. One of the three caregivers in the case group under age 25 had moderately severe depression while none of the caregivers over age 25 had moderately severe depression.

**Table 5:** Association of anxiety and depression in case group caregivers

	p-values
Caregiver age with anxiety	0.031
Health changes with anxiety	0.04
Consanguinity with anxiety	0.029
Caregiver age with depression	0.001

**Table 6:** Association of anxiety and depression in control group caregivers

	p-values
Family support with anxiety	0.005
Family support with depression	0.021

Another positive association was found between caregiver health changes and severe anxiety. One of the eight caregivers in the case group who reported changes in their health suffered severe anxiety, while two of the remaining 52 caregivers in the case group who had no change in their health had severe anxiety.

Another association was found between consanguinity and severe anxiety. Three of 24 caregivers without consanguineous marriages suffered from severe anxiety while none of the 36 caregivers in consanguineous marriages had severe anxiety.

## Discussion

The prevalence of depression in the caregivers of children with diabetes was higher in the case group than in the control group, and this finding is similar to those of other studies. One study in the United States found that an average of 33.5% of parents report distress at the diagnosis of diabetes in their child, while 19% of parents reported distress one to four years after diagnosis [9].

In our study, 56.7% of caregivers of children with diabetes had mild depression, 41.7% had moderate depression, and 1.7% had moderately severe depression. The only variables showing an independent relationship with depression in these caregivers was the age of the caregiver (p = 0.001). The rate of depression is higher among caregivers aged 25 to 40 years compared to those under 25 and over 40 years old.

Variables showing a positive independent relationship with anxiety were caregiver age (p = 0.031), health changes (p = 0.04), and consanguinity (p = 0.029). Delamater et al. reported certain characteristics of children and their parents which predispose them to increased difficulties in diabetes management [10]. Hanna et al. reported the presence of other health problems (e.g., asthma or eating disorders), poor school attendance, learning disabilities, and emotional and behavioral disorders (e.g., risk-taking behaviors) in the child resulting in irresponsible behavior and depression [11].

Factors associated with depression and anxiety can be controlled and managed to decrease the occurrence of depression in these caregivers. Those caregivers who can maintain their child’s glycemic control within reference ranges are prone to have a higher level of stress. Therefore, it is essential that health care providers assess caregivers’ stress regardless of the child’s apparent glycemic control [12]. Further studies are needed to determine which factor has greater effects in causing depression and anxiety among the caregivers.

## Conclusions

Caregivers of children with diabetes have a higher incidence of anxiety compared with caregivers of children without diabetes. However, caring for children with or without diabetes does not seem to be associated with depression. Health care providers should be aware of the differences in parenting stress related to caring for children with long-term illnesses and consider ways to help improve the well-being of these caregivers.
